# Ryugu asteroid sample return provides a natural laboratory for primordial chemical evolution

**DOI:** 10.1038/s41467-023-38518-1

**Published:** 2023-05-30

**Authors:** Yasuhiro Oba, Yoshinori Takano, Jason P. Dworkin, Hiroshi Naraoka

**Affiliations:** 1 Institute of Low Temperature Science (ILTS), Hokkaido University, N19W8 Kita-ku, Sapporo, 060-0819 Japan; 2 Biogeochemistry Research Center (BGC), Japan Agency for Marine-Earth Science and Technology (JAMSTEC), Natsushima, Yokosuka, 237-0061 Japan; 3 Solar System Exploration Division, NASA Goddard Space Flight Center, Greenbelt, MD 20771 USA; 4 Department of Earth and Planetary Sciences, Kyushu University, 744 Motooka, Nishi-ku, Fukuoka, 819-0395 Japan

**Keywords:** Astrobiology, Asteroids, comets and Kuiper belt

## Abstract

The samples returned from near-Earth asteroid (162173) Ryugu provide a pristine record of the 4.6 billion years since the birth of the Solar System. The Hayabusa2 initial analysis team has integrated a range of analytical techniques to investigate Ryugu’s organic chemistry. Here, we highlight their latest findings, the potential questions which may be answered, and provide an overview of new prospects in the decade to come.

## Quest for “natural laboratories” of prebiotic organic chemistry

Astrobiologists have hypothesized that organic compounds in exogenous materials such as carbonaceous meteorites and comets could have made a significant contribution to prebiotic chemistry on the young Earth^1,2^. Therefore, the organic chemical compositions of carbonaceous asteroids may have much potential for deciphering the pathway(s) toward our own origin. However, meteorites collected after landfall quickly become contaminated by Earth’s ubiquitous biology, making it difficult and sometimes impossible to isolate the truly prebiotic signal (e.g., refs. ^3, 4^).

The study of extraterrestrial organic molecules entered a new era once pristine material was remotely sampled at a primitive body and returned to Earth, allowing for extensive laboratory analysis^5^. However, previous sample returns from the volatile deleted stony asteroid (25143) Itokawa^6^, were organic poor; and the comet 81P/Wild 2^7^ samples were irreversibly altered during collection (from high-energy impacts with the aerogel capture medium), meaning further campaigns were necessary.

Hyabusa2 collected samples from two touchdown sites on the near-Earth carbonaceous (C-type) asteroid (162173) Ryugu with minimal alteration^8^, returning 5.4 g of material to Earth on the 6th December 2020. This presents a valuable opportunity to analyse primordial organic molecules in this carbonaceous asteroid and answer scientific questions such as:

- What was Ryugu’s role in the history of the Solar System?
- What are the origins of the organic-constituent volatile elements (C, N, H, O, and S)?
- What do the isotopic compositions of primitive volatile elements tell us?
- How do primitive volatile elements record primordial chemical evolution on the asteroid?
- What mechanisms do prebiotic molecules form by?
- Why does terrestrial life favor one of the chiral isomers (e.g. left-handed for amino acids)?
- How did the interaction of water, organic matter, and inorganic minerals affect the evolution of indigenous molecules?

**Fig. 1:**
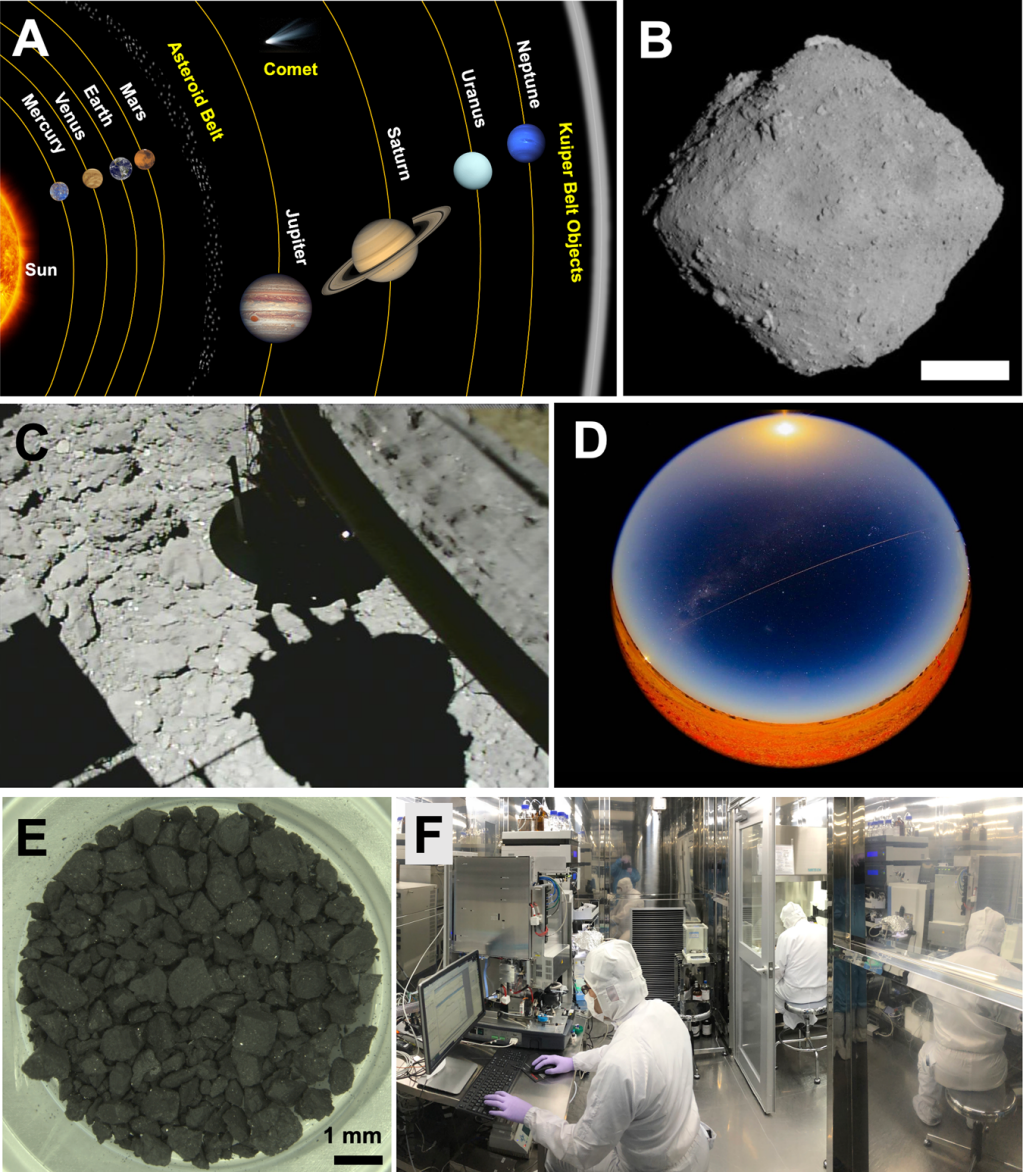
The carbonaceous asteroid Ryugu in the Solar system and exploration for chemical evolution of prebiotic organic molecules by the Hayabusa2 sample return mission. **A** Location of the asteroid belt in the inner Solar system based on the definition of International Astronomical Union (IAU). **B** Global photograph of Ryugu with the ONC-T from 20 km taken on 30-June-2018 (Credit: JAXA, Univ. Tokyo, Kochi Univ., Rikkyo Univ., Nagoya Univ., Chiba Inst. Tech., Meiji Univ., Univ. Aizu, AIST). The white scale bar indicates 200 m. **C** 1st touchdown operations on Ryugu with CAM-H imaging on 22-February-2019, 07:28 (JST), The capture image was just before touchdown during the descent at the altitude of ~4.1 m. (Credit: JAXA). Please see ref. ^8^. **D** Hayabusa2 sample capsule entering the Earth’s atmosphere on 6-December-2020 (Credit: JAXA, photo taken in Australia). **E** Ryugu sample under a microscope for A0106 ref. ^11^. **F** Precise organic chemical analysis and molecular surveys in a clean room (Kyushu Univ.).

Therefore, a careful workflow of sample recovery, sample return, and high-precision organic analysis of the freshest carbonaceous asteroid sample, without uncontrolled exposure to the Earth’s atmosphere^9^ and biosphere^10^_,_ is a critical mission for organic astrochemistry^6,11,12^ (Fig. 1).

The returned Ryugu samples were allocated to the initial analysis teams, including the soluble organic molecule (SOM) sub-team^8,11^. The SOM team extracted organic compounds from ~10 mg of material, which had not been exposed to Earth’s atmosphere, in ultra-clean laboratories (Fig. 1). Both non-destructive and destructive analyses were conducted to evaluate Ryugu’s organic molecules in sequential solvent extracts.

## Pristine bulk profiles and indigenous organic molecules at Ryugu

For bulk profiles, elemental analysis and isotopic ratio measurements were performed for carbon (C), nitrogen (N), hydrogen (H), sulfur (S), and pyrolytic oxygen (O); these are the main atomic components in organic molecules of biological interest. Analyses revealed that ~22 wt% of Ryugu is composed of these volatile light elements^10,12^. Methanol-extracted solutions from the Ryugu samples were analysed for comprehensive molecular profiles. Non-targeted analysis detected a wide variety of ~20,000 different organic molecular species, with molecular weights of up to 700 Daltons^11^.

The first targeted analyses have identified indigenous amino acids, a nucleobase (uracil), vitamer (nicotinic acid), monocarboxylic acids (including acetic acid), alkylamines, and polycyclic aromatic hydrocarbons in solvent extracts^11,13–15^. The spatial distributions and relationships of organic species and inorganic minerals have also been established in a single grain from Ryugu^16^. It has further been demonstrated that sulfur-bearing anionic organic species may have played an essential role in the molecular evolution of prebiotic organic material on this asteroid^17^.

**Fig. 2:**
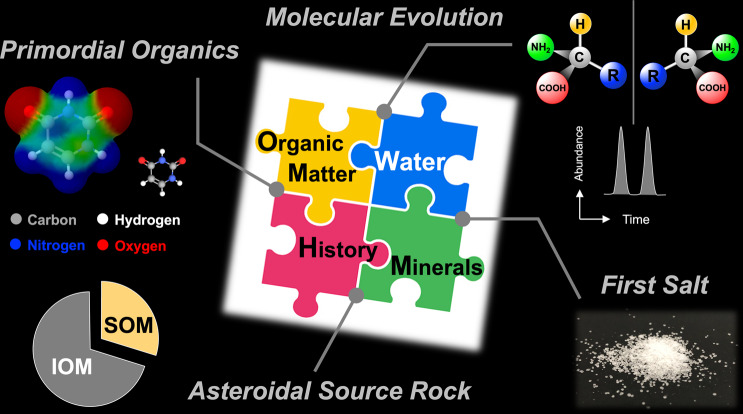
Exploring carbonaceous asteroid from multi-approaches of organic astrochemistry. Pyrimidine nucleobase (uracil) and the intramolecular electrostatic potential are shown as a representative primordial organic molecule^13^. Chiral amino acids and the chromatographic enantiomer separation are shown as one of primary science targets^11,15^. The carbonate, sulfate, and halide salts formed by the interaction of water and minerals will gives us an insight into indigenous aqueous alteration processes^17^. Both soluble and insoluble organic matter (SOM and IOM) are important organic components in the asteroid parent body^11,18^. The SOM and IOM pie charts do not show a strict quantitative relationship between the two fractions.

Insoluble organic matter (IOM) comprises the main portion of the organic carbon in Ryugu samples^18^. The IOM fraction of the carbonaceous asteroid may serve as a reservoir for complex, highly altered, macromolecular organic matter, but this is highly recalcitrant and hard to analyse. The simultaneous acquisition of chemical data for both SOM and IOM is an important analytical approach for future sample return missions (Fig. 2). Eventually, we may be able to provide high-resolution information on how complex organic molecules evolved on asteroids through the interaction of silicate minerals and soluble organic matter^19^. In this context, the dynamics of spatial homogeneity and heterogeneity is also an important perspective^16^. The spatial analyses of organics in the Ryugu samples demonstrate that the distribution of organic species is just as variable as that in carbonaceous meteorites, suggesting this may represent the real distribution of organics in the Solar System.

## Ryugu: an examination of molecular symmetry breaking

As Ryugu most resembles a CI-type meteorite, we simultaneously analyzed a sample of the representative Orgueil meteorite (Denmark Natural History Museum) using the same procedures as for Ryugu^11,13,14^. Orgueil has demonstrated that an extraterrestrial preference for the l-isomer of the amino acid isovaline exists^19^_,_ and other meteorites have shown enantiomeric excesses of other species (as reviewed by refs. ^20, 21^). This infers a contribution from extraterrestrial amino acids to the origin of homochirality on Earth. Hence, organic chemists around the world have been eagerly watching to see if amino acids also exhibit this effect at Ryugu.

Both protein forming and non-protein amino acids were found at lower abundances in Ryugu than Orgueil. Amino acids with stereoisomer structures, however, show no preference for this ‘handedness’ in Ryugu that is been observed in Orgueil and other meteorites^11,15^. Thus, the mystery on the origin of homochirality for terrestrial biology remains uncertain. Hopefully future studies, including further sample return efforts, will reveal the true origin of this chirality.

## Ryugu and further decadal perspectives

**Fig. 3:**
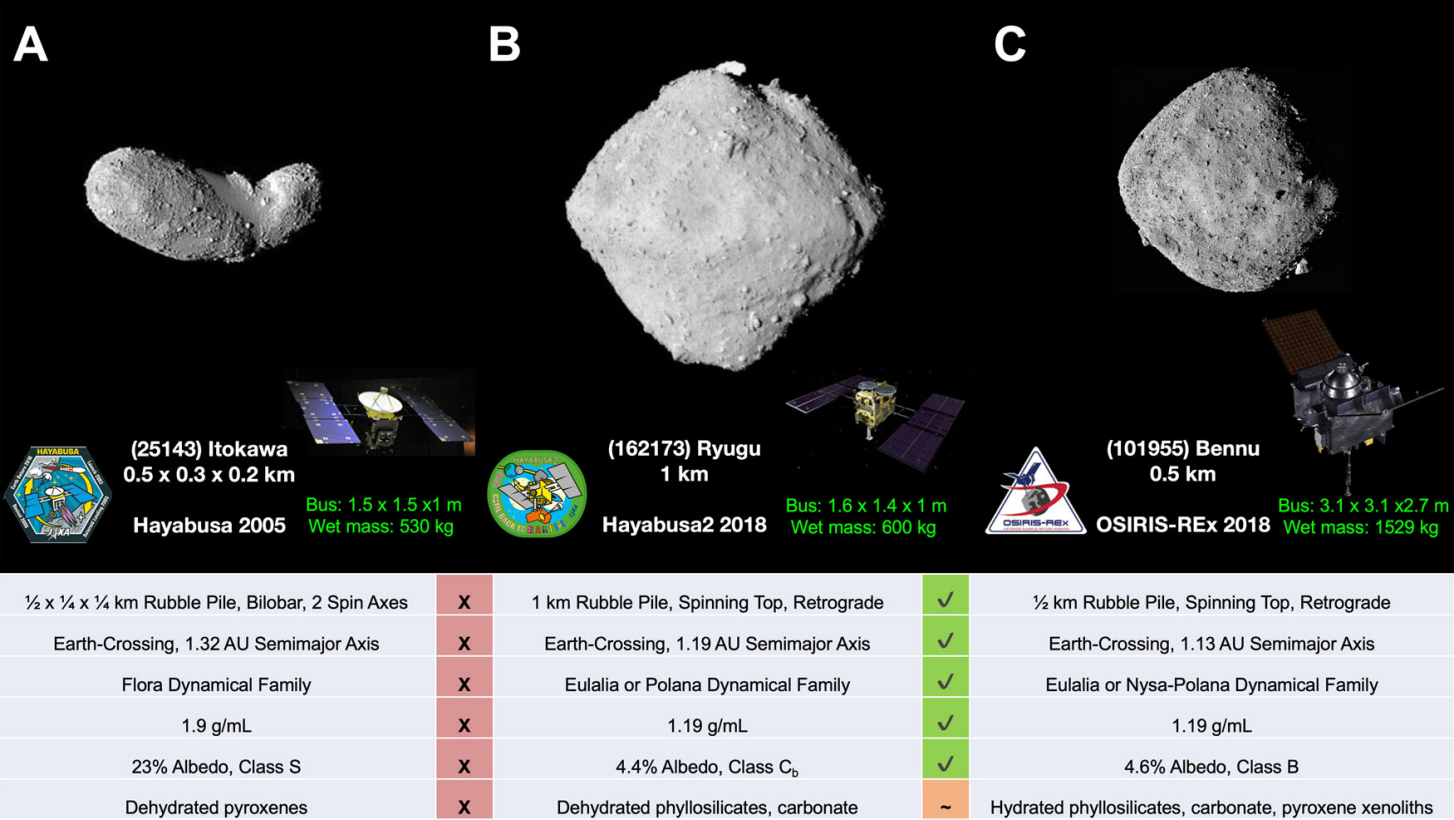
Comparison of asteroid sample return missions. **A** Itokawa and Hayabusa mission. The photograph of Itokawa, the shooting date on 1-March-2007 (Itokawa photo, spacecraft image, and mission logo all credit: JAXA). **B** Ryugu and Hayabusa2 mission. The photograph of Ryugu, the shooting date on 30-June-2018 (Credit: JAXA, Univ. Tokyo, Kochi Univ., Rikkyo Univ., Nagoya Univ., Chiba Inst. Tech., Meiji Univ., Univ. Aizu, AIST, spacecraft image and mission logo credit: JAXA). **C** Bennu and OSIRIS-REx mission (Bennu photo, spacecraft image and mission logo credit: NASA). The basic properties of the objects for size, density, albedo, composition, with spacecrafts for mission durations are shown.

Ryugu’s initial analyses are only the beginning of a new quest for pristine chemical evolution. Following the Hayabusa2 project, other sample-return missions are ongoing or in development. NASA’s OSIRIS-REx (Origins, Spectral Interpretation, Resource Identification, Security, Regolith Explorer) mission is targeting a B-type carbonaceous asteroid (101955) Bennu^22^_,_ returning approximately 250 g of sample to Earth on the 24 September 2023_._ Bennu shares many properties with Ryugu (Fig. 3). Both are rubble-pile carbonaceous near-Earth asteroids, but Bennu exhibits a higher degree of hydration and a history of active aqueous alteration^23^. By comparing both asteroids’ primordial organic profiles, we may observe a diversity of co-evolutional interactions with water, organic matter, and minerals; learning what commonalities are shared and what is unique to different carbonaceous asteroids. We may be able to establish how Ryugu’s and Bennu’s differing journeys, after parent body breakup, to the inner solar system have led to divergent chemical evolution and how the dynamics of the early solar system have influenced these changes. Comparing the chemical evolution of Ryugu and Bennu will surely be scientifically significant in answering the big issues on how the early solar system environment directed chemical processes which ultimately led to the birth of life on Earth.

Further missions are in development to collect and return samples from primitive and evolved bodies. The MMX (Martian Moons eXploration) mission under development by JAXA^24^ targets Mars’ moon Phobos for sample return (>10 g). It will collect surface material from the moon which should contain both primitive materials to unlock both Phobos’s origin and likely sample dust lofted from the Martian atmosphere. NASA’s Artemis missions will use astronauts to collect and return material from the potentially volatile-rich lunar south pole region^25^, these samples will contain lunar material, as collected by Apollo, Luna, and Chang’e 5, but also contain ices derived from countless cometary and asteroidal particles. The joint NASA/ESA Mars Sample Return mission will eventually return drill cores collected by NASA’s Perseverance rover^26^ to search for organics and volatiles from the Martian shallow sub-surface environment.

In the coming decades, further sample return missions from the dwarf planet Ceres, the surface of a comet^27^, a main belt asteroid, and Venus^28^, are all envisioned. With a greater volume and variety of extraterrestrial materials available for laboratory study, further details on the composition and origins of organic molecules in the Solar System will be increasingly understood. Over the next decades, we will be able to keep our eyes on these frontlines for primordial organic molecules. What we learn from such a challenge may tell us how we began.
